# Implementation of dCas9-mediated CRISPRi in the fission yeast *Schizosaccharomyces pombe*

**DOI:** 10.1093/g3journal/jkab051

**Published:** 2021-02-22

**Authors:** Ken Ishikawa, Saeko Soejima, Fumie Masuda, Shigeaki Saitoh

**Affiliations:** Department of Cell Biology, Institute of Life Science, Kurume University, Asahi-machi 67, Kurume, Fukuoka 830-0011, Japan

**Keywords:** knockdown, transcription, CRISPR-Cas, CRISPRi, dCas9, fission yeast, biotechnology

## Abstract

Controllable and reversible transcriptional repression is an essential method to study gene functions. A systematic knock-down method using catalytically inactive Cas9 (dCas9) was originally established in bacteria. dCas9 forms a ribonucleoprotein with a small guide RNA and uses it to recognize a specific DNA sequence via Watson-Crick base-pairing. When specifically bound to a targeted DNA, dCas9 impairs RNA polymerase activity and represses transcription of that target gene. This technology, CRISPRi, has been implemented in several organisms, but not in *Schizosaccharomyces pombe* using dCas9. Here, we provide a plasmid that expresses dCas9 and sgRNA in fission yeast. With this plasmid, CRISPRi repressed endogenous gene transcription by as much as 87%. This transcriptional repression method is controllable, reversible, and efficient enough to alter cellular phenotypes. Here, we offer a CRISPRi method to choose proper targeting sequences for transcriptional repression in fission yeast. Implementation of CRISPRi will help to reveal gene functions and to develop tools based on dCas9 technology in *S. pombe*.

## Teaser

Catalytically inactive mutant of Cas9 (dCas9) can repress gene transcription. This gene knockdown method, called CRISPRi, is useful because it can repress an arbitrary target gene only by changing the sgRNA sequence that determines target gene specificity. Here, dCas9-mediated CRISPRi is implemented for the deeply studied model organism fission yeast, and a method to design sgRNAs for efficient CRISPRi is offered. This will facilitate revealing gene functions and developing tools based on dCas9 technology in *Schizosaccharomyces pombe*.

## Introduction

Clustered regularly interspaced short palindromic repeat—CRISPR associated (CRISPR-Cas) is an adaptive immune system in bacteria and archaea against viruses and plasmids (Doudna and Charpentier 2014). The natural type II CRISPR-Cas system requires three components, Cas9 DNA endonuclease, crRNA (CRISPR RNA), and tracrRNA (transactivating crRNA) to cleave a specific target DNA. crRNA contains a targeting sequence (formally called a spacer sequence), which is typically 20–30 nt in length and complementary to a specific DNA target to be cleaved (Jinek et al. 2012). tracrRNA is required for maturation of the crRNA and for cleavage activity of Cas9. tracrRNA and crRNA bind with Cas9 protein to form a ribonucleoprotein complex. Specificity of the target DNA is determined by two mechanisms: (1) Watson-Crick base-pairing with a targeting/spacer sequence of a crRNA and (2) recognition by Cas9 of a 2–5-bp PAM (protospacer adjacent motif) sequence on the immediate 3′ side of the targeted, protospacer, DNA sequence (Sternberg et al. 2014).

Artificially generated chimeric RNA composed of the crRNA and the tracrRNA, called single-guide RNA (sgRNA), provides robust DNA cleavage of a specific target by forming a complex with Cas9 protein (Jinek et al. 2012). This makes the type II CRISPR-Cas system easy to handle for applications in biotechnology. Since the CRISPR-Cas9 system is able to cleave a specific DNA sequence by simply altering the targeting sequence of sgRNA, it has been utilized to edit genomic DNA in broad range of model organisms, including fission yeast (Jacobs et al. 2014; Rodriguez-Lopez et al. 2016; Zhang et al. 2018; Hayashi and Tanaka 2019). In fission yeast systems, sgRNA is produced by using an RNA polymerase II-expressed rrk1-hammerhead ribozyme cassette, which creates precisely processed sgRNA in fission yeast cells (Jacobs et al. 2014). In this transcription cassette, the sgRNA is transcribed in precursor form connected to a 5′-leader sequence and a 3′-hammerhead ribozyme. These terminal sequences are removed by endogenous RNase III and the self-cleaving hammerhead ribozyme activity, which results in a mature sgRNA. This mature sgRNA mediates specific DNA recognition and cleavage by Cas9, just as the natural crRNA: tracrRNA does.

While the DNA cleavage activity of Cas9 is utilized for genome editing, catalytically inactive Cas9 (dCas9) is also useful for many applications (Pulecio et al. 2017). Cas9 protein has two DNA endonuclease domains, HNH (or McrA-like) and RuvC-like domains, and each domain confers cleavage activity toward one strand of a target double-stranded DNA. Amino acid substitution of catalytic residues in these domains, D10A for the HNH domain and H840A for the RuvC-like domain, abolishes DNA cleavage activity, while its specific DNA-binding activity is retained (Gasiunas et al. 2012; Jinek et al. 2012). Since dCas9 simply binds to specific locus in a genome without cleaving the DNA, proteins such as transcription regulators or fluorescent proteins, can be tethered to a desired locus by fusing them with dCas9, so that they can modulate transcription or visualize a specific genomic region (Pulecio et al. 2017). Interestingly, even when dCas9 is not fused with any other proteins, it efficiently represses transcription of target genes in bacteria (Bikard et al. 2013; Qi et al. 2013), human cell lines, and budding yeast (Gilbert et al. 2013). Compared with a representative gene knock-down method, such as RNA interference, this technology, known as CRISPRi, has the following advantages: (1) the effect is highly specific to a single target gene. Off-target effects are a disadvantage of RNAi methods, (2) it does not require any endogenous RNAi components; thus, it is applicable to broad range of organisms.

The fission yeast, *Schizosaccharomyces pombe*, is a well-studied model organism. A huge accumulation of knowledge and resources from previous studies make this organism useful to explore eukaryote cell biology. However, traditionally, only a limited number of time-consuming methods for suppressing gene expression have been utilized in fission yeast, such as inserting controllable promoters into target gene loci, or expressing long double-stranded RNAs (Bahler et al. 1998; Raponi and Arndt 2003). Lack of a knock-down method hampers systematic characterization of genes essential for viability. Knock-out of essential genes causes cell lethality; thus, it is virtually inapplicable to study functions of such genes. Isolating conditionally defective alleles, such as temperature-sensitive alleles, is widely used to analyze essential genes, but such alleles are not always obtainable. Besides, transcriptional control utilizing dCas9 is expected to facilitate other applications to engineer synthetic transcriptional networks and metabolic pathways (Nielsen and Keasling 2016; Santos-Moreno and Schaerli 2020). Thus, a systematic knock-down method, such as CRISPRi, which can easily be used in fission yeast, is desirable to understand gene functions and to engineer cellular properties of this organism.

Recently, a CRISPRi method based on the CRISPR-Cas12a system was established in *S. pombe* (Zhao and Boeke 2020). However, to our knowledge, CRISPRi mediated by dCas9 had not been implemented in this organism. Here, we report implementation of CRISPRi using a plasmid that expresses both sgRNA and dCas9 in *S. pombe*. By this means, CRISPRi, greatly repressed several genes in the fission yeast genome. Since the transcriptional repression efficiency of CRISPRi depends on where dCas9 binds to a target gene locus, designing the proper targeting sequence is the main challenge in utilizing CRISPRi (Qi et al. 2013; Gilbert et al. 2014; Lawhorn et al. 2014). This study shows that sgRNAs that bind to the non-template strand at the transcription start site (TSS) or those that bind to the template strand approximately 60–120 bp downstream from the TSS result in the most efficient transcriptional repression by dCas9-mediated CRISPRi in fission yeast.

## Materials and methods

### Media and fission yeast strains

Growth media and yeast culture conditions were as described previously (Moreno et al. 1991). Edinburgh minimal medium 2 (EMM2) contains 2% glucose. Supplements were added to EMM2, if necessary, in the following concentrations: 7.5 mg/L adenine for a low-adenine medium, 212.5 mg/L adenine for an adenine-rich medium (EMM2A), 85 mg/L uracil for a uracil-rich medium (EMM2U), 100 mg/L histidine for a histidine-rich medium (EMM2H). Fission yeast strains carrying plasmids are prepared by transformation of strain sp685 (*h^−^ leu1^−^*) by the standard lithium acetate method (Moreno et al. 1991). Other strains, 972 (*h^−^*), sp168 (*h^−^ ura4^−^*), and sp152 (*h^+^ his5-303*) are from our laboratory collection.

### Plasmid construction

**Figure 1:**
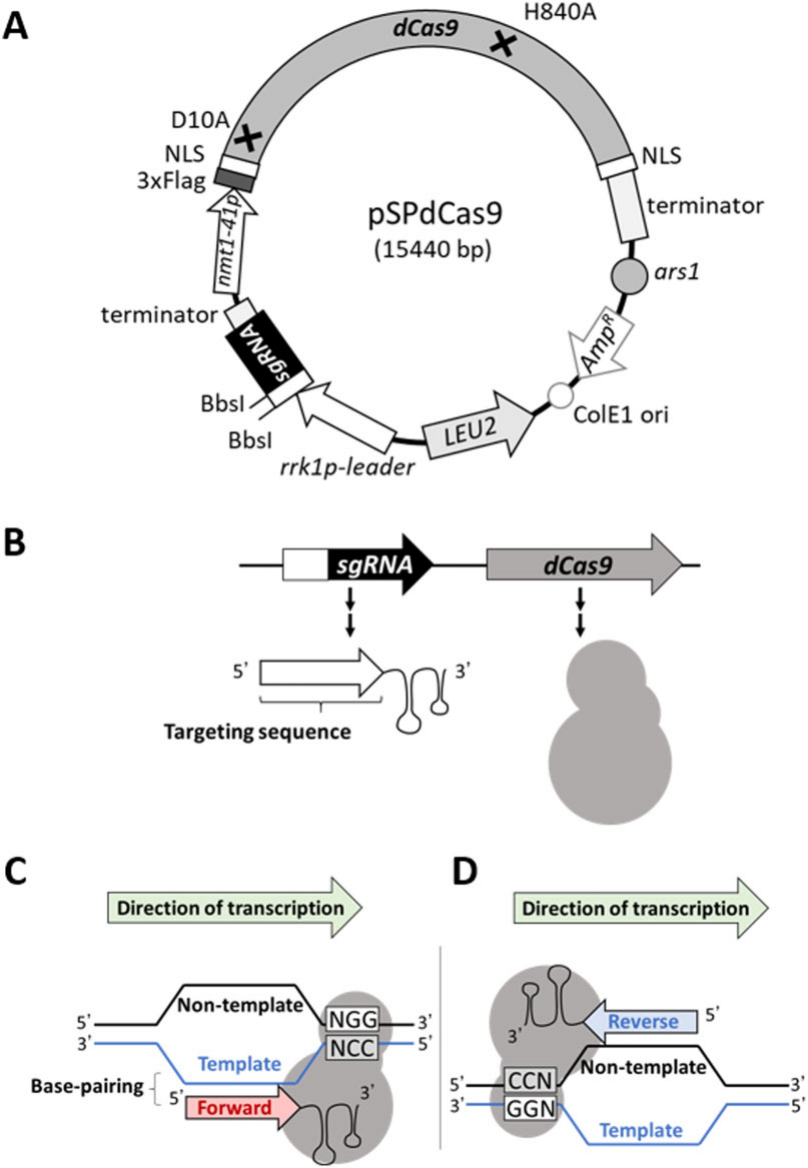
Diagram of the plasmid that expresses dCas9 and sgRNA in *S. pombe.* (A) Plasmid map of pSPdCas9. dCas9, humanized dCas9, originally from *Streptococcus pyogenes*. *nmt1-41p*, a moderate-strength variant of the *nmt1* promoter, transcription of which is inducible. When the targeting sequence is not replaced, pSPdCas9 expresses an sgRNA with a nonsense targeting sequence. Black crossed lines indicate mutations introduced in the dCas9 gene to cause the indicated amino acid substitutions. (B) Components for CRISPRi expressed from the plasmid, pSPdCas9, and its derivatives. sgRNA is first transcribed in precursor form and then processed to become mature sgRNA. The targeting sequence of the sgRNA determines DNA binding specificity of dCas9 (see text for details). An arbitrary targeting sequence can be inserted at the BbsI site of the plasmid (see the Materials and methods section for details). (C, D) Specific DNA binding of dCas9 ribonucleoprotein. Directionality of the targeting sequence determines the orientation of dCas9 bound to the target gene. NLS, nuclear localization signal; 3xFlag, three repeats of the epitope tag Flag; *ars1*, a DNA replication origin of the *S. pombe*; *Amp^R^*, ampicillin resistance gene; ColE1 ori, DNA replication origin for *E. coli*; *LEU2*, the budding yeast leucine biogenesis gene that complements defects of the *leu1* gene in fission yeast; *rrk1p-leader*, a constitutive promoter for the sgRNA gene, including a 5′-leader sequence that is transcribed with and cleaved off in the vicinity of the 5′ end of the sgRNA; sgRNA, the sgRNA gene the targeting sequence of which can be replaced with an arbitrary DNA sequence at the BbsI cleavage sites.

A plasmid, pAH237 (Hayashi and Tanaka 2019), provided by the National Bio-Resource Project (NBRP), Japan, which expresses sgRNA and humanized *Streptococcus pyogenes* Cas9, was modified to express catalytically inactive dCas9 in place of the latter. dCas9 contains two mutations that cause amino acid substitutions at catalytic residues, D10A and H840A, which eliminate DNA endonuclease activity (Gasiunas et al. 2012; Jinek et al. 2012). These mutations are introduced by amplifying part of the Cas9 gene between the two mutations using primers, KI864 and KI865, which contain mutated sequences. The other part of plasmid pAH237 was amplified with another set of primers, KI867 and KI866, which include homologous sequences of the dCas9 gene fragment at their ends. Two of these DNA fragments are connected by DNA recombination to form a full-length dCas9 gene in *E*scherichia *coli* strain SN1187 (Nozaki and Niki 2019). The resulting plasmid, which expresses sgRNA and dCas9, is named pSPdCas9. A detailed map is presented in Figure 1A. The DNA sequence of pSPdCas9 is available as Supplementary Information. The plasmid, pSPdCas9, will be deposited at NBRP (https://yeast.nig.ac.jp/yeast/top.xhtml).

### Modification of the targeting sequence in the sgRNA

Short double-stranded DNA fragments with targeting sequences were inserted into the sgRNA gene on plasmid pSPdCas9, as described previously (Hayashi and Tanaka 2019), with modifications as follows. Targeting sequences were designed using the software, CRISPRdirect, which searched for specific 20-bp targeting sequences with immediately 3′-adjacent PAM sequences (5′-NGG-3′) in the *S. pombe* genome (Naito et al. 2015) (https://crispr.dbcls.jp/). TSS positions, which are utilized to choose and characterize targeting sequences in this report, are based on previously published data (Li et al. 2015). It was reported that a targeting sequence longer than 20 bp did not improve CRISPRi (Qi et al. 2013; Gilbert et al. 2014), and we usually insert a targeting sequence DNA of 20 bp into the plasmid. The designed 20-bp sequences were utilized to synthesize oligo DNAs to form the following double-stranded (ds)DNA (“N” indicates an arbitrary nucleotide residue in the designed targeting sequence, and the annealed regions have complementary sequences). Oligo DNAs were synthesized in a standard desalting purification grade (ThermoFisher Scientific Ltd., U.S.A.).

5′-CACCNNNNNNNNNNNNNNNNNNNN-3′

3′ -NNNNNNNNNNNNNNNNNNNNCAAA-5′

Each of the 20-µM oligo DNAs was annealed in an annealing buffer (10 mM Tris-HCl (pH 8.0), 50 mM NaCl, 1 mM EDTA) by incubation in a thermal cycler under the following conditions: 95°C 2 min, gradual cooling to the melting temperature (Tm) of the targeting sequence at −2°C/min, Tm for 5 min, and gradual cooling to 25°C. The staggered ends of the short dsDNA are complementary to those on the vector fragment, which was prepared by cleaving pSPdCas9 with a restriction enzyme, BbsI. The targeting sequence was inserted into the sgRNA gene by ligating 2.0 pmol of the short dsDNA and 0.03 pmol (0.3 µg) of the vector fragment with DNA ligase, using solution I of the Takara ligation kit ver.2 (Takara Bio Inc., Japan). The resulting solution was introduced into *E. coli* strain DH5α. Transformants were selected on LB plates supplemented with 100 mg/L ampicillin and subjected to liquid culture to purify plasmids. Insertion of the targeting sequence DNA into the plasmid was confirmed by PCR amplifying a DNA fragment using one of the oligo DNAs used to prepare the insert dsDNA and primer KI908. Another primer set (KI907 and KI910) that amplifies a DNA fragment specific to pSPdCas9 was utilized to detect the original plasmid that was not modified. The procedure described above usually provides about 70% plasmids with the expected structure. Lists of primer sequences and plasmids are available in the Supplementary Information.

### Induction of dCas9 expression

Transcription of the dCas9 gene is controlled by an inducible promoter, *nmt1-41p*, transcription of which is inhibited in the presence of 15 μM thiamine in the medium (Forsburg 1993). Before inducing dCas9 expression, fission yeast cells carrying the plasmid were grown on EMM2 plates supplemented with 20 µM thiamine and nutrients that complement auxotrophy of the target gene (these media are referred as EMM2A + T20, EMM2U + T20, or EMM2H + T20), at 33°C for about 24 h. To induce dCas9 expression, thiamine was removed by extensive washing of the cells—a small patch, about 3 mm diameter, of grown cells was washed five times with 1 mL of sterilized water and resuspended in 500 µL of sterilized water. Of the washed cell suspension, 200 µL was added to 2 mL of EMM2 with appropriate nutrients, but lacking thiamine (EMM2A, EMM2U, or EMM2H). Cells were then incubated at 33°C for 6–8 hours with shaking. The resulting culture was diluted to 9.4 × 10^4^ cells/mL in 10 mL of EMM2 medium with appropriate nutrients, but without thiamine, followed by incubation at 33°C for 20 h with shaking.

### RT-qPCR

After induction of dCas9 expression as described above, fission yeast cells were recovered by centrifugation at 2000 rpm for 2 min at room temperature. Cell pellets were washed with 1 mL of sterilized water and frozen in liquid nitrogen. Frozen cells were stored at −80°C until total RNA preparation. Total RNA was prepared by phenol extraction (Lyne et al. 2003). cDNAs were prepared using a reverse transcriptase ReverTra Ace qPCR RT Kit (Toyobo Co., Ltd., Japan) under conditions described in the product manual. cDNAs were quantified with real-time PCR (LightCycler, Roche, Ltd., Germany) and FastStart Essential DNA Green Master Mix (Roche, Ltd., Germany). mRNA was quantified relative to *act1^+^* mRNA. Primer sequences for qPCR are listed in the Supplementary Information.

### Data analysis

Percent repression as indicated in Figure 4C was calculated as follows. mRNA levels were measured by RT-qPCR as described above from at least three biological replicates. mRNA levels of *ade6^+^* or *ura4^+^* were converted to relative values by dividing them by the mean of mRNA levels in nonsense controls. Reciprocals of resulting relative mRNA levels were defined as the repression index, *R*_x_ (repression index of a targeting sequence “x”). Percent repression was calculated as shown in the equation below. Mean (*R*_ns_) indicates a mean of repression indexes from nonsense (ns) controls, and this is a background value of the repression index. The mean (*R*_max_) in the CRISPRi for *ade6^+^* is a mean of repression indexes from results of the most effective targeting sequence a4, while that for *ura4^+^* was calculated from results of targeting sequence u17. As indicated in the equation, % repression is a percentage of maximum net repression indexes. Figure 4C shows % repression described above as mean ± standard deviation from at least three biological replicates.

$$\%\;\mathrm{Repression}\;=\;\frac{R_{x}-\mathrm{Mean}\;(R_{\mathrm{ns}})}{\mathrm{Mean}\;\left(R_{\max}\right)-\mathrm{Mean}\;(R_{\mathrm{ns}})}\;\times 100$$

### Phenotypic reversibility test

Reversibility of phenotypes caused by dCas9 was tested by re-repressing dCas9 expression after induction. dCas9 expression was induced as described above, and cells were grown on EMM2 plates with low adenine to show the red color caused by *ade6^+^* gene repression. To re-repress dCas9 expression, an aliquot of cells grown on EMM2 low-adenine medium was streaked on EMM2A + T20 plates, and was incubated at 33°C for 24 h. A small patch of cells was grown in 2 mL of EMM2A + T20 broth at 33°C for 6–8 hours with shaking. The resulting culture was diluted to 9.4 × 10^4^ cells/mL in 2 mL of EMM2A + T20 broth, followed by incubation at 33°C for 20 h with shaking. This cultured cell suspension was streaked onto an EMM2 low-adenine plate to test *ade6^+^* expression, followed by incubation at 30°C for 4 days.

## Results

### CRISPRi mediated by dCas9 efficiently represses transcription of the *ade6*^+^ gene in *S. pombe*

**Figure 2:**
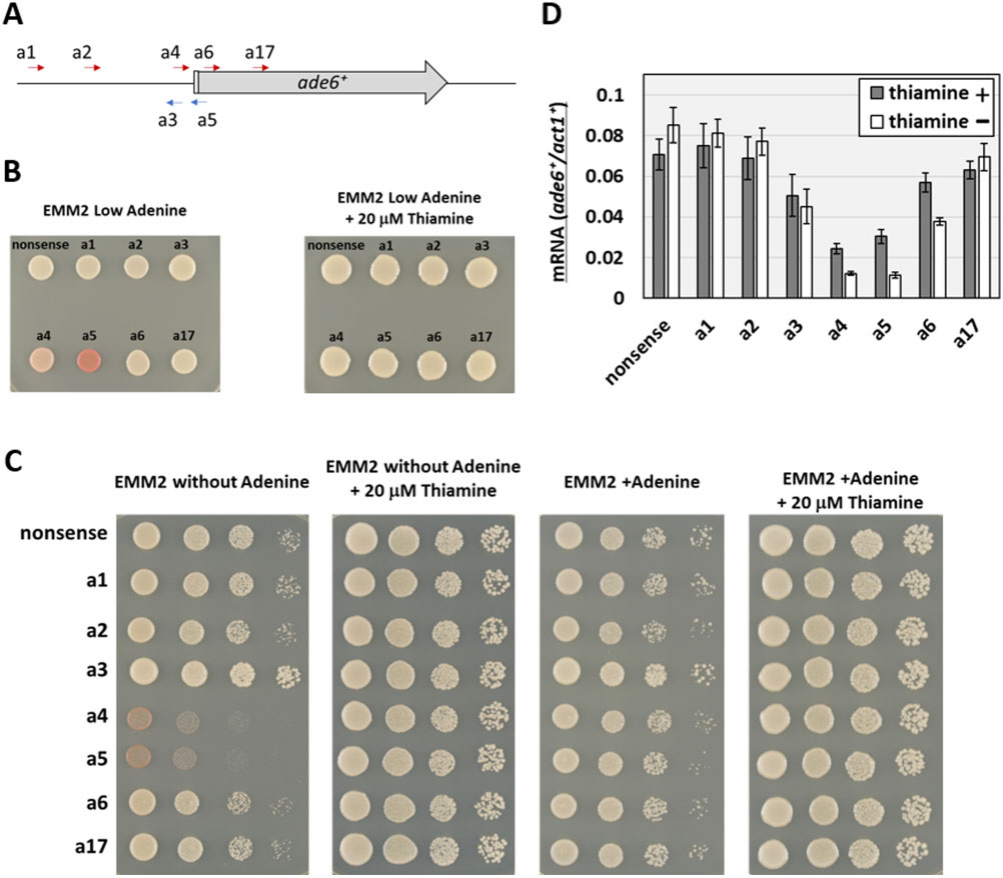
dCas9-mediated CRISPRi represses transcription of *ade6^+^*. (A) Diagram of the designed targeting sequences. Red and blue arrows indicate targeting sequences of sgRNAs that base-pair with the template and non-template strand of the targeted DNA, respectively. PAM sequences (5′-NGG-3′) are situated immediately to the 3′-side of the arrowheads (not shown in the diagram). A light gray box indicates the 5′UTR, of which the left end is the TSS. (B) Color assay for *ade6^+^* expression. Yeast cells carrying a plasmid that expresses dCas9 and sgRNA with the indicated targeting sequence were spotted on the medium (see the Materials and Methods for details). (C) Serial dilution (10-fold) spot test growth assays of fission yeast cells bearing pSPdCas9 plasmids with the indicated targeting sequences on differentially supplemented media as shown. (D) Quantification of *ade6^+^* mRNA. mRNA of *ade6^+^* and *act1^+^* was quantified by RT-qPCR. Relative mRNA amounts of *ade6^+^* normalized by that of *act1^+^* in the presence of sgRNAs containing the indicated targeting sequences are presented. Gray bars indicate relative mRNA levels in the medium supplemented with 20 µM thiamine, while white bars indicate those in the medium without thiamine. Results show means ± standard deviation from three biological replicates.

To test whether dCas9 can repress gene transcription in fission yeast, we modified plasmid pAH237 (Hayashi and Tanaka 2019), which expresses sgRNA and Cas9 protein in *S. pombe*, so that it expressed dCas9 instead of Cas9 (Figure 1A). sgRNA and dCas9 expressed from the modified plasmid pSPdCas9 form a ribonucleoprotein, which binds to a specific DNA site through Watson-Crick base-pairing of the sgRNA and the targeted DNA, when the proper targeting sequence is contained in the sgRNA (Figure 1, B–D). In the plasmid, pSPdCas9, the sgRNA gene contains an 18-bp cloning site as a nonsense targeting sequence, which is not homologous to any sequence in the *S. pombe* genome. Expression of dCas9 protein was placed under control of the *nmt1-41* promoter, from which transcription is induced upon removal of thiamine from the medium. To repress transcription of a model target gene, *ade6^+^*, short (20-bp) sequences derived from the *ade6*^+^ gene (a1–6 and a17, Figure 2A) were ligated to the sgRNA gene on the plasmid, pSPdCas9, as its targeting sequence, through substitution of the nonsense targeting sequence by a standard DNA cloning method (see the Materials and methods section for details). Resulting plasmids were introduced into *S. pombe* wild type cells, and the expression level of *ade6*^+^ was estimated from the color of colonies formed by the cells. Since impaired expression of *ade6*^+^, which encodes phosphoribosylaminoimidazole carboxylase, required for adenine production, results in accumulation of a reddish intermediate on EMM2 medium supplemented with a low concentration (7.5 mg/L) of adenine, the expression level of the *ade6^+^* gene can be estimated by the color of transformed colonies.

While fission yeast cells harboring plasmid pSPdCas9, which contains the nonsense targeting sequence, formed white colonies, cells carrying a plasmid with the *ade6^+^* targeting sequence a5 formed red colonies, when expression of dCas9 was induced by removal of thiamine (Figure 2B, left). Similarly, a plasmid with the targeting sequence a4 made colonies pink. These results indicate that expression of the *ade6*^+^ gene was effectively repressed by CRISPRi. Judging from colony color, among seven sgRNAs tested, two sgRNAs containing a targeting sequence (a4 or a5) close to the TSS efficiently repressed expression of *ade6*^+^ (Figure 2, A and B). This result is consistent with previous reports showing that sgRNAs binding close to TSSs repress gene transcription effectively in other model organisms (Qi et al. 2013; Gilbert et al. 2014). On the other hand, when dCas9 expression was repressed by 20 µM thiamine in the medium, all cells formed white colonies, indicating that CRISPRi was dependent on dCas9 expression (Figure 2B, right). Thus, CRISPRi, as implemented here, is controllable by removal or addition of thiamine. Notably, expression of dCas9 alone did not appear to interfere with cell proliferation. The control plasmid, pSPdCas9, did not affect the color or growth of colonies (Figure 2B).

In addition to the colony color assay, repression of *ade6*^+^ expression was confirmed by testing adenine auxotrophy (Figure 2C). CRISPRi with sgRNAs containing the targeting sequence, a4 or a5, impaired colony formation on EMM2 medium without adenine, whereas colony formation was not impaired on media supplemented with thiamine. This is consistent with results of the colony color assay (Figure 2B). Colony formation was recovered by supplementation with adenine (Figure 2C), indicating that impaired colony formation was caused by lack of adenine, rather than by cytotoxicity of the sgRNAs and dCas9.

To quantify the degree of transcriptional repression of the *ade6^+^* gene by CRISPRi, the amount of *ade6^+^* mRNA was measured with RT-qPCR (Figure 2D). CRISPRi with the targeting sequence a4 reduced *ade6^+^* mRNA level to 14% of the control, and that with the targeting sequence a5 reduced the level to 13%. This degree of repression is comparable to effect of dCas9-mediated CRISPRi in a human cell-line (Lawhorn et al. 2014). With targeting sequence a4, a5, or a6, reduction in the *ade6^+^* mRNA level was observed even in the presence of thiamine, although the degree of *ade6^+^* transcription repression was lower than in the absence of thiamine. As the presence of thiamine does not completely inhibit transcription from the *nmt1-41p* promoter (Forsburg 1993) (Supplementary Figure S1), leakage of *dCas9* transcription repressed *ade6^+^* transcription to levels such that cell growth and colony colors were not affected. Collectively, these results indicated that dCas9 is able to repress *ade6^+^* transcription when the proper sgRNA is provided and that transcriptional repression is controllable and sufficiently efficient to induce *ade6*-deficient phenotypes (*i.e.*, adenine auxotrophy and red colony formation).

### The effect of dCas9-mediated CRISPRi is reversible in fission yeast

**Figure 3:**
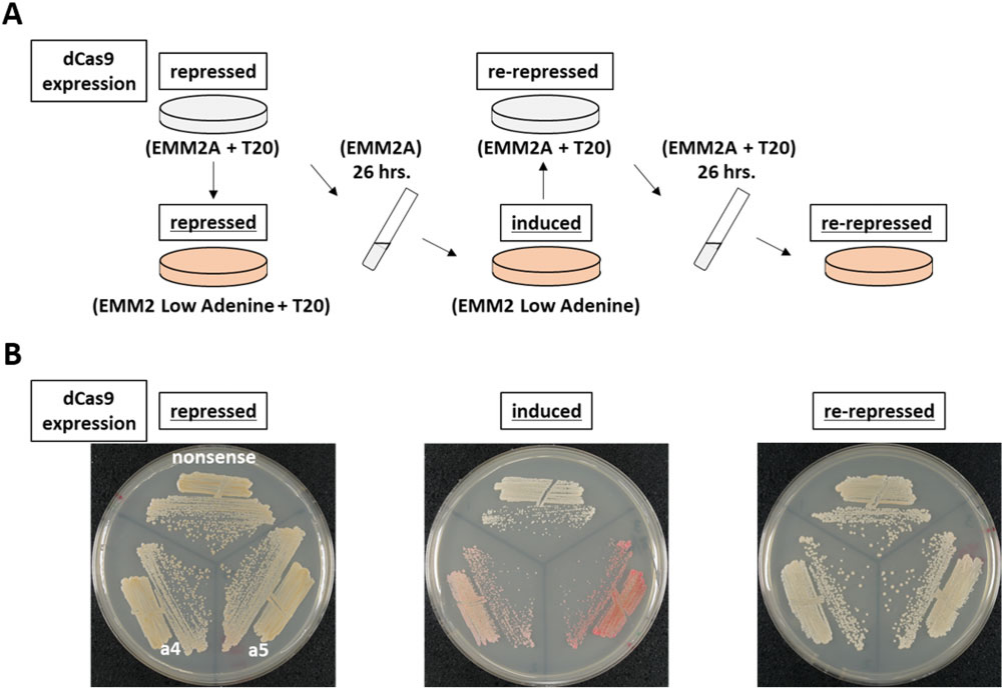
Transcriptional repression by dCas9-mediated CRISPRi is reversible. (A) Diagram of the experimental procedure. dCas9 expression was sequentially controlled by removal or addition of 20 µM thiamine (T20). *ade6^+^* expression was estimated by colony color at the steps indicated with orange plates that contain media with a low concentration of adenine. Results are shown in panel B. Circular plates indicate cultures on solid media. Test tubes indicate culture in liquid media. (B) Results of reversibility of transcriptional repression by CRISPRi. Yeast cells carrying pSPdCas9 plasmids containing the indicated targeting sequences were grown as shown in panel (A) and streaked on media plates corresponding to orange plates as indicated above (panel A). EMM2A, adenine rich medium; T20, 20 µM thiamine.

As described below, *ade6^+^* repression by dCas9-based CRISPRi is reversible when dCas9 expression is sequentially controlled (Figure 3A). This is an advantage compared with a conventional gene knock-out method, in which the genome is irreversibly altered. Cells carrying pSPdCas9 plasmids expressing dCas9 and sgRNA with a targeting sequence (nonsense, a4, or a5) formed white patches on low-adenine solid medium supplemented with 20 μM thiamine (Figure 3B, Left). These strains were grown in liquid medium without thiamine and cultured for one day to induce dCas9 expression. Resulting cells were streaked on medium without thiamine to test the colony color phenotype. Upon induction of dCas9 on solid medium lacking thiamine, cells with the targeting sequence, a4 or a5, formed reddish colonies (Figure 3B, middle). When these colonies were subjected to another round of solid and liquid culturing in the presence of thiamine to repress dCas9 expression again, colonies turned white after being re-streaked on solid medium with thiamine (Figure 3B, right). This result indicated, as expected, that our CRISPRi system does not repress gene transcription by altering the genomic DNA sequence and that re-repression of dCas9 expression had recovered transcription of *ade6*^+^.

### sgRNAs that bind close to a TSS are preferred by CRISPRi in fission yeast

**Figure 4:**
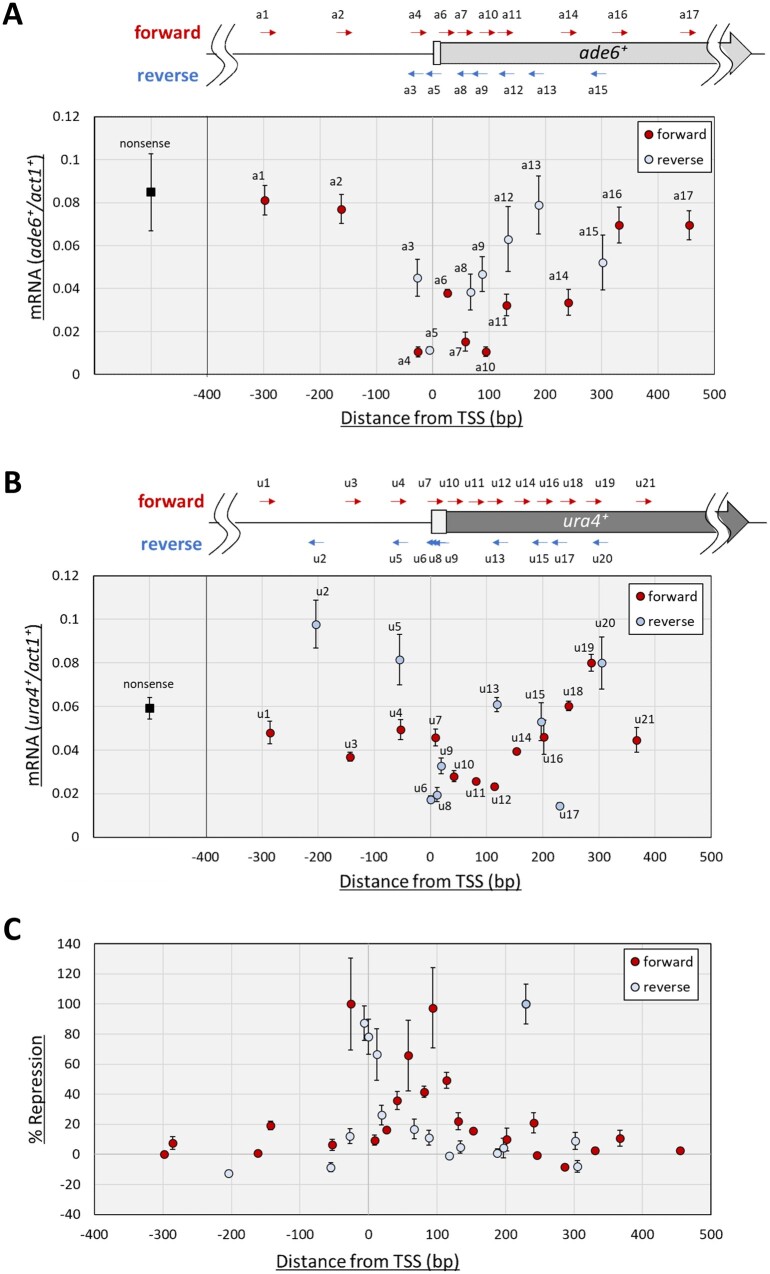
sgRNA targeting position and transcriptional repression efficiency. (A) Quantification of *ade6^+^* mRNA by RT-qPCR with sgRNAs that have the indicated targeting sequence. Results are shown as means ± standard deviation from at least three biological replicates. For a1-a6 and a17, the same data as in Figure 2D (Thiamine−) are shown. For a4, additional biological replicates were included in analysis. (B) Quantification of *ura4^+^* mRNA by RT-qPCR with sgRNAs that have the indicated targeting sequence. Results are shown as means ± standard deviation from three biological replicates. (A, B) Diagrams are labeled as Figure 2A. (C) Percentage of repression observed in *ade6^+^* and *ura4^+^* are merged. Percentages were calculated as values relative to the mean of repression observed with the most effective targeting sequence in each of the genes, for which percentages are defined as 100% (see the Materials and methods section for details). This calculation is based on data presented in panels A and B. Results are shown as means ± standard deviation from at least three biological replicates. (A–C) Red circles indicate data for forward targeting sequences, and blue circles indicate those of reverse targeting sequences. Nonsense, nonsense targeting sequence; a1-a17 and u1-u21, targeting sequences.

DNA-binding specificity of dCas9 is determined by a targeting sequence in sgRNA. Understanding the relationship between transcriptional repression efficiency and the position to which dCas9 binds is important for designing sgRNAs that are effective for CRISPRi. It was reported that dCas9 binding close to the TSS of a target gene efficiently represses transcription in bacteria and human cell-lines (Bikard et al. 2013; Qi et al. 2013; Gilbert et al. 2014). Consistent with these reports, transcription of *S. pombe ade6*^+^ was also effectively repressed by sgRNAs containing targeting sequences, a4 or a5, which bind in close proximity to the *ade6^+^* TSS. To confirm that targeting sequences close to the TSS efficiently repress gene transcription in fission yeast by CRISPRi, more sgRNAs were generated to target the *ade6*^+^ gene and another model target gene, *ura4*^+^. Using these sgRNAs, transcriptional repression efficiency was examined in relation to proximity to the TSS (Figure 4). On *ade6^+^*, some sgRNAs targeting −26 bp to 94 bp from the TSS (the distance from the TSS to the center of the 20-bp targeting sequences, a4, a5, a7, and a10) showed efficient repression, by which the *ade6^+^* mRNA level was reduced to 13–18% of that in the nonsense control (Figure 4A). sgRNAs with targeting sequences farther from the TSS were less effective. This suggests that sgRNAs targeting a region close to the TSS are preferred for effective repression. A similar tendency was also observed with the other gene, *ura4^+^* (Figure 4B). Among the tested sgRNAs, those targeting 0 to 114 bp from the *ura4^+^* TSS (u6, u8, u11, and u12) efficiently reduced the *ura4^+^* mRNA level to 28–43% of the control (Figure 4B). These results collectively indicate that designing sgRNAs targeting approximately −30 to +100 bp from the TSS is preferred for efficient repression in fission yeast. Transcriptional repression of *ura4^+^* gene by dCas9-mediated CRISPRi was not sufficient to cause uracil auxotrophy and 5-FOA resistance (Supplementary Figure S2).

### Preferred directionality of targeting sequences for dCas9-mediated CRISPRi in fission yeast

In addition to the distance from the TSS, directionality (forward or reverse strand) of targeting sequences also affected repression efficiency (Figure 1, C and D). Targeting sequences of sgRNAs can be designed as the “forward targeting sequence,” which is identical to that of the forward (non-template) strand of a targeted gene locus, and as the “reverse targeting sequence,” which is identical to that of the reverse (template) strand. Targeting sequences mediate DNA recognition of dCas9 through Watson-Crick base-pairing interaction with a targeted DNA strand; therefore, sgRNAs with the forward targeting sequence bind to the template strand of the gene, whereas those with the reverse targeting sequence bind to the non-template strand (Figure 1, C and D) (Qi et al. 2013). Consequently, directionality of the targeting sequence determines the relative orientation between a dCas9 molecule bound to a DNA and the direction of targeted gene transcription. Interestingly, the directionality of the targeting sequence affected transcriptional repression efficiency in *E. coli*, in which sgRNAs with reverse targeting sequences resulted in stronger repression than those with the forward targeting sequences, when they are designed to bind downstream of the TSS (Bikard et al. 2013; Qi et al. 2013).

Directionality of the targeting sequence may affect CRISPRi efficiency, even in fission yeast, but the effect appeared to be opposite to that observed in *E. coli*. When sgRNAs targeted downstream from the TSS (about 60–120 bp), forward targeting sequences showed stronger repression than those with reverse targeting sequences in *S. pombe* (Figure 4). In the case of *ade6^+^*, three pairs of targeting sequences, closely situated in opposite directions (a7-a8, a9-a10, and a11-a12), were tested for transcriptional repression efficiency, and the forward targeting sequences showed more efficient repression than did the others (Figure 4A). A similar result was obtained with *ura4^+^*. Forward targeting sequence, u12, caused stronger repression than the closely situated targeting sequence of the reverse strand, u13 (Figure 4B). These results indicate that forward targeting sequences are preferable 60–120-bp downstream from the TSS in *S. pombe*.

In contrast, in a region close to the TSSs (−30 to +15 bp), the effect of directionality was not clear. In this region of *ura4*^+^, reverse targeting sequences (u6, u8, and u9) showed stronger repression than a forward targeting sequence (u7) (Figure 4B). Similarly, on *ade6^+^*, a reverse targeting sequence, a5, showed efficient repression (Figure 4A). However, contrary to this, in the case of a closely situated pair of targeting sequences, a3 and a4, the forward targeting sequence (a4) showed stronger repression than the reverse targeting sequence (a3). Collectively, reverse targeting sequences may tend to repress gene transcription more efficiently in proximity to the TSS, although further accumulation of examples is required to reveal general tendencies of directionality close to TSSs (−30 – +15 bp). Candidates for a targeting sequence are less available upstream of the TSS than in coding sequence regions. The PAM sequence of dCas9 is GC-rich (5′-NGG-3′), while the upstream region of a TSS is usually AT-rich. Some targeting sequences resulted in even higher transcription than in the control (Figure 4B, u2, u5, u19, and u20). Perhaps these sites are involved in inhibition of *ura4^+^* transcription.

### Design of targeting sequences for dCas9-mediated CRISPRi in fission yeast

Analysis of CRISPRi on *ade6^+^* and *ura4^+^* (Figure 4, A and B) suggests two preferred designs of targeting sequences. When results of *ade6^+^* and *ura4^+^* were converted to relative repression efficiencies and merged into a single panel (Figure 4C), two distinct peaks of repression efficiency became evident. Among results from forward targeting sequences, those ∼60–120-bp downstream from the TSS showed the highest efficiency, whereas reverse targeting sequences formed a narrow peak around −5 bp from the TSS. According to these tendencies, we hypothesized that (1) forward targeting sequences that bind ∼60 - 120 bp downstream from a TSS and (2) reverse targeting sequences that overlap or almost overlap the TSS are preferable for dCas9-mediated CRISPRi in fission yeast.

According to these tendencies, we designed additional targeting sequences for the *his2^+^* or *his7^+^* genes—(i) forward targeting sequences 60–120 nucleotides downstream from the TSS or (ii) reverse targeting sequences that overlap or almost overlap the TSS (Supplementary Figure S3). Two out of four tested targeting sequences for *his2^+^* gene, h2-1 and h2-4, successfully reduced the mRNA level to less than half (22–43%) that of the control. The most efficient repression of the *his2^+^* gene by h2-1 caused histidine auxotrophy. Comparable repression for *his7^+^* was also observed—two out of four tested sequences, h7-1 and h7-2, reduced the *his7^+^* mRNA level to less than half (39–49%) of that in the control, though this repression was not sufficient to cause histidine auxotrophy. Thus, the design strategy described above, (1) and (2), can be utilized as a guideline to choose targeting sequences for efficient transcriptional repression by dCas9-mediated CRISPRi in fission yeast.

## Discussion

Here, we showed that dCas9-based CRISPRi is able to repress gene transcription effectively in fission yeast. By dCas9-mediated CRISPRi, the expression level of *ade6^+^* was reduced efficiently enough to cause deficient adenine metabolism, which results in accumulation of a reddish intermediate and adenine auxotrophy. This repression was controllable and reversible by addition or removal of thiamine. In addition, our results of CRISPRi on *ade6^+^* and *ura4^+^* resulted in a protocol to choose targeting sequences for efficient transcriptional repression in fission yeast.

Design principles for targeting/spacer sequences of sgRNAs for dCas9-based CRISPRi have been explored in bacteria and human tissue cultures (Bikard et al. 2013; Qi et al. 2013; Gilbert et al. 2014). Even among such distantly related organisms, it is commonly observed that gRNAs that bind in the vicinity of a TSS caused more efficient repression than those binding to sites distant from the TSS. This was also true in *S. pombe*, as revealed in this study. Another aspect of the targeting sequence that may affect transcriptional repression efficiency is directionality. In *E. coli*, a set of experiments to repress expression of a reporter gene revealed that reverse targeting sequences, which bind to the non-template strand, caused stronger repression than forward targeting sequences, when they are downstream of the TSS (Bikard et al. 2013; Qi et al. 2013). Such preferences in strand directionality of targeting sequences were not observed in a large-scale analysis of gene repression by dCas9 in human cell lines (Gilbert et al. 2014). These results suggest that the effect of directionality of the targeting sequence may differ among organisms and/or genes. Based on the present results, using four independent model genes, *ade6*^+^, *ura4*^+^, *his2*^+^, and *his7*^+^, we predict that sgRNAs with reverse targeting sequence at the TSS and forward targeting sequences 60–120-bp downstream from the TSS repress gene transcription efficiently in *S. pombe*. Further exhaustive analyses, such as genome-wide analyses of sgRNAs, may be required for more precise prediction and design of the most effective sgRNA sequences for CRISPRi in fission yeast.

Although this study provides a protocol for choosing targeting sequences of sgRNAs, targeting sequences at potentially the most suitable position/orientation are not always available for dCas9-mediated CRISPRi due to the restriction that the targeted DNA must contain the PAM sequence (5′-NGG-3′) at the 3′ terminus of the base-pairing region. Genetic mutations of Cas9 relaxed the specificity of the PAM sequence (Hu et al. 2018). Utilizing such mutant dCas9 would help to design sgRNAs in cases in which sequences matching the PAM sequence of normal Cas9 are not found in a suitable position/orientation of a gene. The other approach to bypass the limitation of PAM is to employ another CRISPR-Cas system with different PAM specificity. Recently, it was reported that a type V CRISPR system, CRISPR-Cas12a, is applicable for CRISPRi in fission yeast (Zhao and Boeke 2020). Since Cas12a has a T-rich PAM, 5′-TTTV-3′ (V is A, G, or C), Cas12a and Cas9, for which PAM is G-rich and short 5′-NGG-3′, complement each other to cover wide variety of target sites.

Endogenous chromatin proteins may also affect CRISPRi efficiency. It was reported that nucleosomes impede CRISPRi by dCas9s fused with a transcription repressor domain in human cell lines and budding yeast (Horlbeck et al. 2016; Smith et al. 2016). Their analysis, based on repression of thousands of genes, revealed that effective targeting sequences are situated in regions that have low nucleosome occupancy. Moreover, dCas9 binding to DNA was eliminated by histone proteins upon nucleosome assembly *in vitro* (Horlbeck et al. 2016). In this study, repression efficiency as a function of distance from the TSS showed two peaks (around −5 bp, and 60–120 bp) and a drop between the peaks (10–60 bp from the TSS, Figure 4C). Interestingly, a genome-wide micrococcal nuclease protection analysis revealed that the highest peak of nucleosome occupancy is located around 50 bp downstream from TSSs, followed by another peak around 200 bp (∼152-bp interval), in fission yeast (Givens et al. 2012). Thus, peaks of nucleosome occupancy and repression efficiency appear mutually exclusive. These results suggest that nucleosomes may impede CRISPRi mediated by dCas9, even in fission yeast. Another chromatin factor that possibly affects CRISPRi efficiency is the FACT (FAcilitates Chromatin Transcription) complex, which is conserved in eukaryotes, including fission yeast. The FACT elevates turnover frequency of Cas9 binding to DNA, and this leads to increased DNA cleavage by Cas9, but to decreased histone modification by dCas9 fused with a histone modifier (Wang et al. 2020). These results suggest that the FACT complex may inhibit CRISPRi by dCas9 in fission yeast. Endogenous factors that affect the dynamics of dCas9 may alter CRISPRi efficiency.

dCas9 is utilized not only for CRISPRi but also for many other applications by fusion with functional proteins, to engineer DNA sequence-specific enzymes, to visualize specific genomic loci, to pull-down/enrich specific DNA fragments, and so on (Pulecio et al. 2017). Understanding basic characteristics of how dCas9 without protein fusion behaves in cells will facilitate development of such biological tools. Implementation of controllable CRISPRi and protocols to choose proper targeting sequences provided in this study will facilitate future studies to understand gene functions in fission yeast.

## Data and reagent availability

The plasmid pSPdCas9 will be available at NBRP (https://yeast.nig.ac.jp/yeast/top.xhtml). Other strains and plasmids are available upon request. All data necessary to confirm conclusions of the article are present within the text, figures, and tables. Supplementary files are available online. Supplementary Figure S1 contains results of *dCas9* mRNA quantification. Supplementary Figure S2 contains results of uracil auxotrophy and 5-FOA sensitivity tests on yeast cells subjected to dCas9-mediated CRISPRi for *ura4^+^* gene. Supplementary Figure S3 contains results of transcriptional repression of *his2^+^* or *his7^+^* gene by dCas9-mediated CRISPRi and histidine auxotrophy test. Supplementary Table S1 contains a list of oligo DNAs used in this study. Supplementary Table S2 contains a list of plasmids used in this study. Supplementary File S1 contains DNA sequence of the plasmid pSPSdCas9. Supplementary material available at figshare: https://doi.org/10.25387/g3.14038109.
